# Prevalence of hyperglycemia in masters athletes

**DOI:** 10.7717/peerj.13389

**Published:** 2022-05-13

**Authors:** Mike Climstein, Joe Walsh, Kent Adams, Trish Sevene, Tim Heazlewood, Mark DeBeliso

**Affiliations:** 1 Clinical Exercise Physiology, Faculty of Health, Southern Cross University, Bilinga, Queensland, Australia; 2 Exercise and Sport Science Exercise, Health & Performance, Faculty Research Group, Faculty of Health Sciences, University of Sydney, Sydney, Australia; 3 Sport Science Institute, Sydney, New South Wales, Australia; 4 Kinesiology Department, California State University Monterey Bay, Seaside, CA, United States of America; 5 Department of Kinesiology and Outdoor Recreation, Southern Utah University, Cedar City, CA, United States of America

**Keywords:** Fasting plasma glucose, World Master Games, Exercise, Obesity, Veteran athlete, Prevention, Risk factors, Physical activity

## Abstract

**Background:**

Ageing is associated with decreased physical activity, obesity and subsequently an increased risk of developing type 2 diabetes mellitus (T2dm). Master athletes (MA) have initiated exercise or sport later in life or pursued a physically active lifestyle for an extended period. Subsequently, MAs have been proposed as a model of successful ageing as this active lifestyle is associated with health benefits including decreased health risk of chronic diseases and a reduction in premature mortality. Given long-term physical activity/exercise has previously been shown to be protective against hyperglycemia, a risk factor for T2dm, it is plausible that MA may have protective benefit against developing hyperglycemia. Therefore, the aim of this study was to investigate the prevalence of hyperglycemia via fasting plasma glucose (FPG) in MAs competing at the World Masters Games (WMG).

**Methods:**

This cross-sectional, observational survey utilized an online survey using open-source web-based software was used to investigate MAs physiological and medical-related parameters. Over 28,000 MAs competed in the WMG, of which 8,072 MAs completed the survey. Of these MAs, a total of 486 (males 277, females 209; range 27 to 91 years, mean age 55.1 ± 10.2 years) attained recent pathology results which included FPG which was subsequently analyzed for this study. FPG and other outcome variables were compared between genders and to the Australian and United States general population.

**Results:**

Mean FPG for MAs was 5.03 mmol (±1.2, 95% CI [4.9–5.1] mmol) with majority (75.5%) of MAs reporting a normal (<5.5 mmol) FPG, followed by pre-diabetes (20.2%, >5.51 to <5.99 mmol) and abnormal (4.3%, >7.0 mmol). There was no significant difference (*P* = 0.333) in FPG between genders however, males had a slightly higher (+2.1%) FPG as compared to females (5.08 ± 1.2 mmol (95% CI [4.9–5.22] mmol) versus 4.98 ± 1.1 mmol (95% CI 4.8-5.1 mmol)). The majority of males (71.8%) and females (80.3%) were classified with a normal FPG. With regard to an abnormal FPG level, only 4.0% of males and 4.9% of females were classified abnormal which was suggestive of undiagnosed T2dm. With regard to age by decade, there was no significant difference (*P* = 0.06–1.00) between age groups and no relationship between the MAs’ age and FPG (*r* = .054, *P* = 0.24). As a group, MAs had a significantly lower FPG as compared to the Australian (−3.2%, *P* = 0.005) and United States general populations (−13.9%, *P* < 0.001).

**Conclusions:**

Most, however not all, MAs were found to have normal glycaemia, with only a small percentage indicating a risk of developing T2dm (i.e., impaired fasting glucose) and a smaller percentage identified with an abnormal FPG, suggestive of T2dm. These findings suggest MAs appear to be at low metabolic risk for developing T2dm based upon FPG and the physical activity/exercise they complete as MAs may indeed be protective against hyperglycemia whilst maintaining an active lifestyle.

## Introduction

Type 2 diabetes mellitus (T2dm) is recognized as a worldwide epidemic and a significant strain on healthcare systems. Jhan and colleagues in 2020 Khan et al. (2020) reported that approximately 462 million individuals were affected by T2dm worldwide, which equated to over six percent of the world’s total population at that time (6,059 per 100,000). These authors predicted that the global T2dm prevalence will increase to 16.8 percent by 2030 and a further increase to 29.8 percent by 2040. Within Australia, the Australian Institute of Health and Welfare (AIHW) Australian Institute of Health and Welfare (2020) estimated there are approximately 1.2 million Australians with T2dm, representing 5.3 percent of the total population in 2018. The AIHW reported that T2dm was more common in Australian men (6.1%) versus women (4.6%) and higher as age increased (*e.g.*, 10.9% in males aged 55–64 and 7.7% in females of the same age range). Furthermore, diabetes contributed to 11 percent (underlying or associated) of all deaths in 2018.

Elevated blood glucose has also been associated with a high burden of deaths. The World Health Organization (WHO) has previously reported that approximately 43 percent of all deaths before the age of 70 were attributed to a high blood glucose and occurred prematurely. This represented 1.6 million deaths worldwide (World Health Organization, 2016).

Hyperglycemia, defined as an elevated blood glucose ≥ 7.0 mmol (Mouri & Badireddy, 2021) is recognized as an independent risk factor for T2dm. It has been reported that approximately eight to 24 percent of individuals with hyperglycemia develop T2dm, based upon their fasting blood glucose level (Fonseca, 2009). Those individuals with a FPG ranging from 6.1 mmol to 6.9 mmol were found to develop T2dm over an average of 29 months (Fonseca, 2009). Asymptomatic hyperglycemia presents in many individuals as they age and increases the susceptibility of a number of age-related diseases such as cognitive decline, cardiovascular disease (Chia, Egan & Ferrucci, 2018) and T2dm. The Australian Institute of Health and Welfare (Australian Institute of Health and Welfare, 2020) has identified that hyperglycemia was responsible for the total burden of each type of diabetes (type 1 diabetes, type 2 diabetes, gestational diabetes), 60 percent of the burden of chronic kidney disease and 6.7 percent of the coronary heart disease burden.

Older adults are at greater risk of developing T2dm due to a number of factors which includes genetics, body mass index (BMI), lifestyle, particularly individuals inactive and aging influences (Lee & Halter, 2017). Hyperglycemia leads to the development of T2dm when there is an imbalance between glucose production (*i.e.*, hepatic glucose production) and glucose intake (*i.e.*, dietary intake of carbohydrate) versus glucose uptake, which is primarily by skeletal muscle (Richter & Hargreaves, 2013). Specific to ageing, hyperglycemia leads to the development of T2dm via impaired beta-cell function in the pancreas and impaired beta-cell adaptation to insulin resistance. Ageing has also been proposed to be associated with a reduced capacity to regenerate pancreatic beta-cells and subsequently leads to hyperglycemia (Rankin & Kushner, 2009).

One mechanism shown to mitigate hyperglycemia is physical activity and exercise. Exercise, both aerobic and resistance training, extracts blood glucose from the systemic circulation as an energy source. This mechanism of controlling glucose levels is quite effective as glucose uptake has been reported to be up to 50-fold with exercise (Sylow et al., 2017; Rose & Richter, 2005) and is comparable to some oral antidiabetic medications(Boulé et al., 2001; Huang, Fang & Tang, 2021). This uptake in glucose by active musculature during exercise is associated with both acute and chronic exercise, regardless of the mode (aerobic or resistance training). For example, Oguri et al. (2009) investigated the effects of a brief, acute bout of aerobic exercise (cycling) on glucose uptake. They reported that glucose uptake was significantly augmented within 15 min of initiating the cycling exercise. Similarly, Adams (2013) conducted a review of brief, high-intensity exercise and reported that a single session in individuals with T2dm improved the postprandial blood glucose for 24 h post exercise. Resistance training studies that were 2-weeks in duration resulted in a 13 percent reduction in blood glucose 48 to 72 h post-exercise, the average blood glucose was reduced by 13% at 48 to 72 h post-exercise and glucose transporter type 4 (GLUT4) increased by 369%.

Not surprisingly, exercise is well recognized as an effective, evidence-based therapy in the prevention and non-pharmacological treatment of hyperglycemia, prediabetes and T2dm (Gillett et al., 2012; Raveendran, Chacko & Pappachan, 2018; Kanaley et al., 2022; Sigal et al., 2007). An additional benefit to completing physical activity and exercise is its simultaneous effectiveness on a number of T2dm related comorbidities such as hypertension, dyslipidemia, obesity and premature death (Cunningham et al., 2020). It is therefore plausible, given the extent of evidence supporting the benefits of physical activity and exercise on blood glucose, that active individuals, particularly those who are regularly active would have a protective effect against the development of hyperglycemia and subsequent development of T2dm.

Masters athletes (MA) are generally older individuals who complete physical activity and systematic exercise training which exceeds the recommended guidelines and are involved in organized sporting competition with similar-aged individuals (Brun, 2016). The minimum age for MA competing is sports specific, although MAs are generally aged 30 years and older (International Masters Games Association, 2020). The International Masters Games Association estimated there are approximately 30,000 male and female MAs from over 100 countries competing in their World Masters Games (WMG) quadrennial sports tournament. Previously, MAs have been reported to be healthier than the general population and therefore this group has been proposed to represent a model of successful ageing (Climstein et al., 2019; Hawkins, Wiswell & Marcell, 2003; Geard et al., 2017; Tanaka, Tarumi & Rittweger, 2019). Although MAs are not well investigated, there are a number of studies on MAs which have identified the health benefits on a number of biomedical health determinants and risk factors (Australian Institute of Health and Welfare, 2012). For example, MAs have been reported to have significantly lower risk for obesity as assessed by BMI (Climstein et al., 2019; Walsh et al., 2013a; Walsh et al., 2011b; Walsh et al., 2011d; Walsh et al., 2013b; Walsh et al., 2018; Walsh, Heazlewood & Climstein, 2018; Debeliso et al., 2014) and lipids (Climstein et al., 2018).

There has however, only been a single study which has investigated glycaemia in MAs. Our research group (Climstein et al., 2011a) investigated the FPG of 216 golden oldies rugby festival (GORF) MAs (mean age 51.2 ± 8.0 years). The GORF is an international rugby competition held biannually and open to all MAs aged 35 and over. The mean FPG of the GORF MAs was reported as 5.7 mmol (SD ± 2.9). Of interest, the GORF MAs had a reported prevalence of diabetes (type 1 and type 2) significantly higher than the general Australian population mean at that time (7.5% *vs* 4.0%, respectively) despite a mean FPG within the normal, non-diabetic range. However, the investigators did not report on other findings relative to glycaemia such as pre-diabetes or abnormal FPG (suggestive of T2dm) as the focus of that study was the incidence of chronic diseases and lipid profiles in the GORF participants. It was therefore the purpose of this study to select MAs from our WMG data set (*n* = 8, 070 participants, collected in 2009) who provided FPG and related risk factor data for analyses. We hypothesized that MAs would report lower FPG levels than compared to the general population.

## Materials & Methods

### Study design

This research utilized a cross-sectional, observational survey epidemiological design which investigated the demographics, risk factors and fasting plasma glucose of participants at the WMG. Approval to conduct this study was granted by the Bond University Human Research Ethics Committee (BUHREC RO1682) in accordance with the ethical standards of the Helsinki declaration of 1975, revised in 2008 (World Medical Association, 2013).

The WMG organizing committee approved this study upon the proviso that the survey would only be provided to the participants in an online, web-based format to minimize any potential disruption to the participants whilst preparing or competing at the WMG. Subsequently, we developed an on-line, web-based questionnaire (LimeSurvey, an open-source survey application written in PHP that is available under the GNU general public license) to deliver the survey to participants. The survey questions consisted of array, single choice, multiple choice, dropdown choices and numerical input. We utilized in-built filters, tick and dropdown boxes to expediate the survey completion time in an attempt to increase participant numbers.

The survey consisted of three sections: demographics, medical health history and physiological parameters. The survey link initial page provided information about the study with the option to provide electronic informed consent to participate in the study. Those participants who provided informed consent then were provided access to the study.

### Sample

Eligible participants in this study included all WMG competitors (*n* = 28, 676), national (*n* = 20, 089) and international (*n* = 8, 587) who were competing in at least one of the 43 sports during the games period. The WMG organizing committee restricted direct access by the researchers to the competitors and therefore the WMG organizing committee sent an invitation email (and follow-up email invitations) to participate in the study to all registered participants with a valid email address (*n* = 24, 528). Potential participants were advised in the invitation email to obtain information from either their general practitioner or primary care physician, which would be required to complete the survey. This included height, mass, waist circumference, resting blood pressure, lipids (total cholesterol, high density lipoproteins, low density lipoproteins, triglycerides) and fasting plasma glucose (amongst other biomedical markers). Body mass index was calculated from each participants height and mass. Participants were informed that lipid and fasting plasma glucose results were only to be reported if requested by their doctor and completed by a commercial pathology laboratory (as opposed to a desktop/hand-held analyzer or glucometer). The lipid (Climstein et al., 2018; Climstein et al., 2011b) and BMI findings have been reported in separate publications (Walsh et al., 2013a; Walsh et al., 2011b; Walsh et al., 2011d; Walsh et al., 2013b; Walsh et al., 2018; Walsh, Heazlewood & Climstein, 2018; Walsh et al., 2012; Walsh et al., 2011c; Walsh et al., 2011a; Walsh et al., 2011g; Walsh et al., 2011h; Walsh et al., 2011e; Walsh et al., 2011f; Walsh et al., 2013c). Given the large percentage (71.2%) of the World Master Games participants in this data-subset were from Australia, we chose to use data from the WHO data for Australia (World Health Organization, 2021). Additionally we used data from United States (World Health Organization, 2021) and from the National Health and Nutrition Examination Survey (NHANES, *n* = 3, 376) (Centers for Disease Control, 2020) for comparative purposes.

Due to confidentiality concerns, participants were not required to upload biochemical data which may have included pathology or other medical results not pertinent to this study. The survey link initial page provided information about the study with the option to provide electronic informed consent to participate in the study. Those participants who provided informed consent then were provided access to the study.

**Figure 1:**
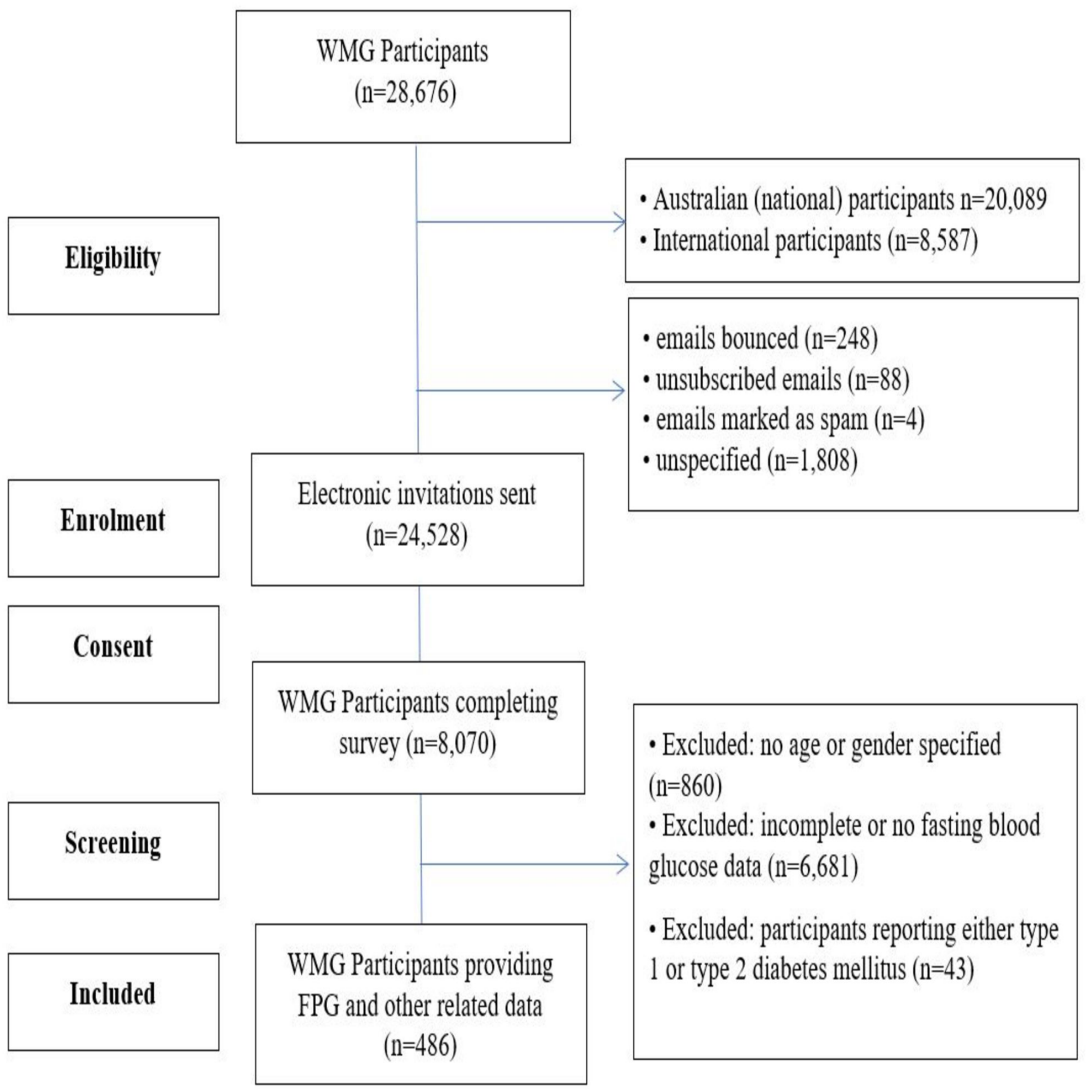
CONSORT flow diagram of participants.

Any participants who reported diabetes mellitus (type 1 diabetes mellitus or type 2 diabetes mellitus) were excluded from this study (=43). Participants with a family history of diabetes mellitus were however, included in this data set. An overview of the participant recruitment for this study is provided in Fig. 1.

### Physiological and biochemical classifications

The classification for BMI was based upon the Royal Australian College of General Practitioners (RACGP) management of type 2 diabetes handbook (Royal Australian College of General Practitioners, 2020) where an underweight BMI was calculated to be <18.5 kg/m^2^, normal BMI was ≥18.5 to <25 kg/m^2^, overweight was a BMI of ≥25.0 to <30 kg/m^2^ and obese was a BMI calculated as ≥30.0 kg/m^2^. The classifications for waist circumference were also based upon the RACGP handbook, however, were gender specific. For males, a normal waist circumference was <94 cm, increased risk of obesity related disorders was a waist circumference of ≥94 cm to <102 cm and a high-risk of obesity-related conditions a waist circumference of ≥102 cm. For females, the normal waist circumference was <80 cm. A female waist circumference associated with an increased risk of obesity related disease was ≥80 cm to <88.0 cm. A female MA with a waist circumference of ≥88 cm was considered to be at very high risk of obesity-related complications (Royal Australian College of General Practitioners, 2020).

The classifications for both resting systolic and diastolic blood pressure (SBP and DBP, respectively) were based upon the most recent American College of Cardiology and American Heart Association recommendations (Flack & Adekola, 2020). The following represent the resting blood pressure categories; normal (SPB < 120 mmHg, DBP < 80 mmHg), elevated (SBP ≥ 120- ≤ 129 mmHg, DBP < 80 mmHg), hypertension stage 1 (SBP ≥ 130- ≤139 mmHg, DBP ≥ 80- ≤ 89 mmHg) and hypertension stage 2 (SBP ≥ 140+ mmHg, DBP ≥ 90 mmHg+). Blood pressure results are presented as resting BP, resting SBP and resting DBP classifications.

Classifications for fasting plasma glucose were made in accordance of the Royal Australian College of General Practitioners screening and diagnosing T2dm in asymptomatic individuals (Royal Australian College of General Practitioners, 2020). A FPG <5.5 mmol was classified as optimal and clinically as diabetes unlikely. A FPG from ≥5.5 to ≤ 6.9 mmol was classified clinically as diabetes possible and a FPG ≥7.0 mmol was classified as diabetes likely, however not diagnostic of T2dm based upon a single FPG test result.

### Statistical analyses

All data was initially analyzed for normality by investigating kurtosis, skewness and QQ plots (visually and analyzed). We also utilized the Kolmogorov–Smirnov test with a Lilliefors significance correction. We assessed heteroscedasticity using Levene’s test for equality of variances. Between gender comparisons were completed using an independent samples *T*-test. Comparisons between MA group data and the World Health Organization data for Australia and the United States and the NHANES was completed using a one-sample *T*-test (2-sided significance). An ANOVA (with Bonferroni post-hoc test) was used to determine if significance existed between age by classifications (age by decade, BMI, waist circumference, BP) and FPG. Bivariate correlations were used to determine if a relationship existed between selected outcome variables and the strength of the relationship. All statistical analyses were performed with the Statistical Package for the Social Sciences (SPSS, ver 28.0.0.0 (190); SPSS Inc, IBM Company, Armonk, NY, USA). Alpha was set *a priori* at *P* < 0.05 to determine statistical significance.

## Results

**Figure 2:**
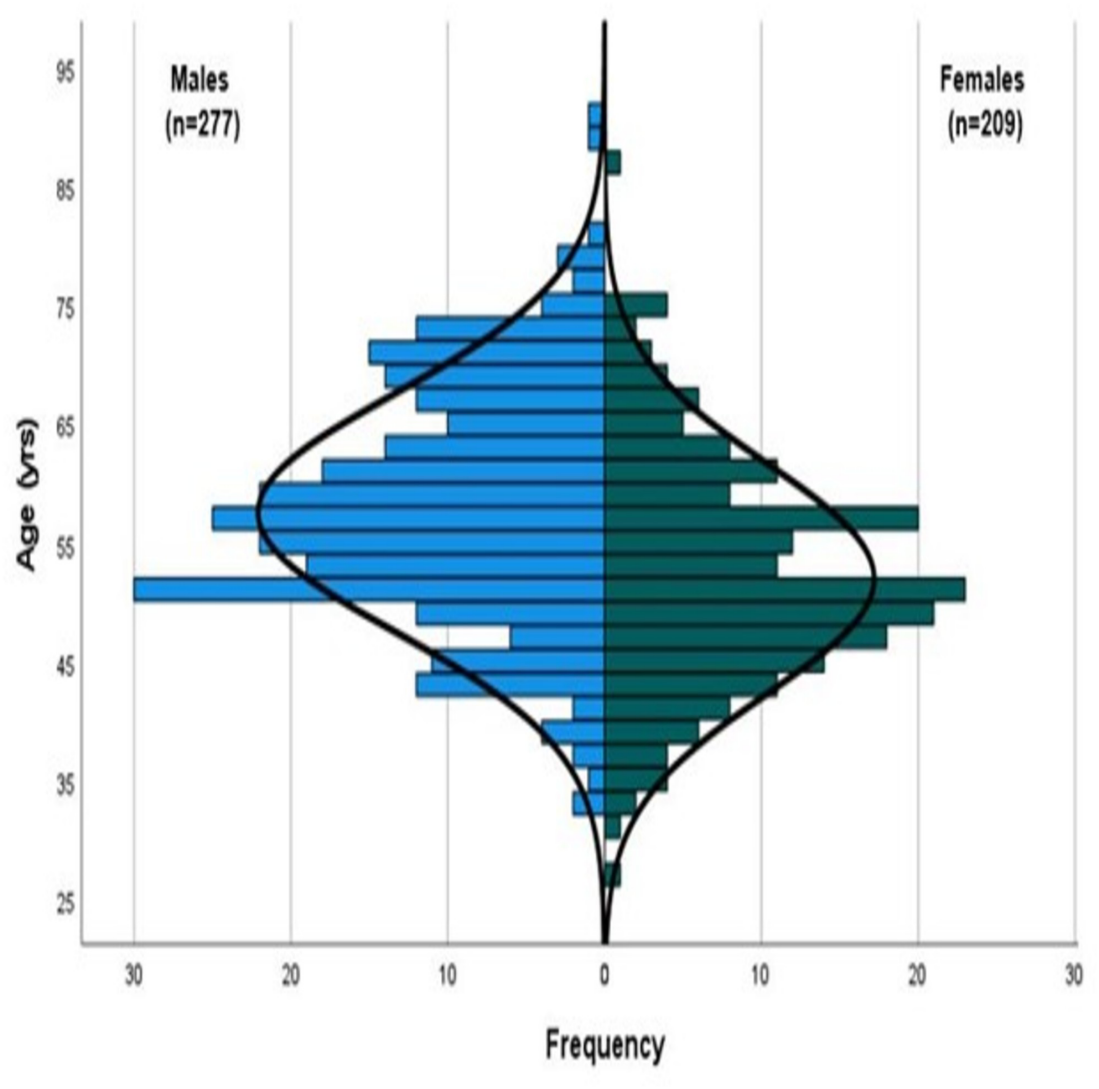
Population pyramid (line of normality illustrated).

Over 28,000 MAs participated in the WMG. Those registrants with a valid email address were sent electronic invitations to participate (*n* = 24, 528). A total of 8,070 (32.9%) of the MAs completed our survey. Of the total participants completing our survey, 486 MAs (males *n* = 277, females *n* = 209), ranging from 27 to 91 years of age (mean age 55.1 ± 10.2 yrs) participated in this aspect of the survey research (Fig. 2).

As a group, the majority of MAs were non-smokers (80.5%) and reported consuming alcohol (81.3%). Of those MAs that consumed alcohol, the mean number of standard drinks per week was 6.3 (±5.4, 95% CI [5.7–6.8] drinks/wk). Male MAs reported consuming significantly more standard drinks per week than female MAs (+37.1%, *P* < 0.001). Of those MAs that reported being current smokers (3.0%), the mean number of cigarettes smoked per week was 83.5 (±19, 95% CI [42–124]), with no difference between genders.

Biometric characteristics identified that males were significantly older (+10.8%, *P* = < 0.001) and heavier mass (+17.4%, *P* < 0.001) than female MAs. Female MAs however, had a similar BMI with a significantly smaller waist circumference (−11.8%, *P* < 0.001). With regard to BMI, the majority of male MAs were primarily classified as overweight (47.4%, ≥25.0 to <30.0 kg/m^2^) whereas female MAs were primarily classified as normal (52.4%, ≥18.5 to <25.0 kg/m^2^). These results are detailed further in Table 1. There was no correlation between MAs age and mass (*P* = 0.41) or age and BMI (*P* = 0.06).

**Table 1:** Demographics and cardiometabolic risk factors of master athletes.

	Group (*n* = 486)	Males (*n* = 277)	Females (*n* = 209)
Age (yr)	55.1 ± 10.2 (54.9–57.0)	57.5 ± 10.0 (57.1–59.7)	51.9 ± 9.6^*^ (51.1–54.1)
Smoking status			
• Non-smoker (%)	80.5	82.5	78.0
• Ex-smoker (%)	16.5	16.5	16.5
• Smoker (%)	3.0	1.1	5.5
Drinking status			
• Non-drinker (%)	17.0	17.5	16.4
• Ex-drinker (%)	1.7	1.8	1.5
• Drinker (%)	81.3	80.7	82.1
Mass (kg)	76.9 ± 14.4 (75.2–77.9)	82.2 ± 11.7 (80.6–83.5)	69.9 ± 14.8^*^ (66.9–71.1)
BMI (kg/m^2^)	25.8 ± 4.3 (25.3–26.1)	25.9 ± 3.4 25.4–26.2)	25.7 ± 5.2 (24.7–26.3)
Classification			
• Underweight (%)	.08	0.8	1.0
• Normal (%)	46.5	41.9	52.4
• Overweight (%)	40.2	47.4	30.6
• Obese (%)	12.5	9.9	16.0
Waist circumference (cm)	85.9 ± 11.3 (84.6–86.8)	89.8 ± 9.7 (88.5–90.9)	80.3 ± 11.2^*^ 78.4–81.8)
Classification			
• Normal (%)	59.1	66.7	48.0
• Increased risk (%)	25.8	22.0	31.5
• High risk (%)	15.1	11.3	20.5
Resting SBP (mmHg)	121.5 ± 12.1 (120.1–122.5)	124.4 ± 10.6 (122.9–125.7)	117.5 ± 11.9^*^ (115.4–119.2)
BP Classification			
• Normal (%)	30.4	17.5	46.3
• Elevated (%)	25.2	27.4	22.6
• HTN stage 1 (%)	34.2	41.9	24.7
• HTN stage 2 (%)	10.1	13.2	6.3
SBP Classification			
• Normal (%)	32.8	19.7	48.9
• Elevated (%)	37.7	41.9	32.6
• HTN stage 1 (%)	20.8	26.9	13.2
• HTN stage 2 (%)	8.7	11.5	5.3
Resting DBP (mmHg)	73.9 ± 8.0 (73.1–74.7)	75.8 ± 7.2 (74.8–76.7)	71.6 ± 8.4^*^ (70.0–72.6)
Classification			
• Normal (%)	63.2	56.0	72.1
• HTN stage 1 (%)	33.5	40.2	25.3
• HTN stage 2 (%)	3.3	3.8	2.6
FPG (mmol)	5.0 ± 1.2 (4.9–5.1)	5.1 ± 1.2 (4.9–5.2)	4.9 ± 1.1 (4.8–5.1)
FPG classification			
• Normal (%)	75.5	71.8	80.3
• Pre-diabetes (%)	20.2	24.2	14.8
• T2dm likely (%)	4.3	4.0	4.9

**Notes.**

Values are mean ± SD (95% confidence interval) and percent (%, where indicated).

* *p* < 0.05.

The majority of male and female MAs waist circumferences (gender specific) (66.7% *vs.* 48.0%, respectively) were classified as normal (male < 94 cm, female < 80 cm) (Royal Australian College of General Practitioners, 2021) (Table 1). There was however, a significant (*P* < 0.01) correlation between age and waist circumference (*r* = 0.174).

Females had a significantly lower resting SBP (−6.2%, *P* < 0.001) and DBP (−5.9%, *P* < 0.001) as compared to male MAs. Additionally, the majority (46.3%) of females were normotensive (<120 mmHg/<80 mmHg) whereas the majority of male MAs reported HTN stage 1 (≥130- ≤ 139 mmHg/≥90 mmHg) resting blood pressure (41.4%) (Table 1).

When specific to resting SBP, the majority (48.9%) of female MAs reported normal classification whereas the majority (41.9%) of male MAs reported an elevated SBP. With regard to resting DBP, the majority of males and females were classified as normal (56.0% *vs* 72.1%, respectively) (Table 1). There was a significant correlation between age and resting SBP (*P* < 0.05, *r* = 0.119) or age and resting DBP (*P* < 0.05, *r* = 0.02).

The mean FPG for the MAs as a group was optimal (5.0 ± 1.2 mmol; 95% CI [4.9–5.1] mmol) with the majority (78.5%) being classified as an optimal (≤5.5 mmol) FPG. This was followed by MAs being classified as having a normal (≥5.51 to ≤6.0 mmol) FPG (8.8%) and pre-diabetic (≥6.1 to ≤6.99 mmol) classification (8.4%). Only a small percentage of MAs (4.3%) were classified as having an abnormal FPG, suggestive of T2dm.

There was no significant difference (*P* = 0.33) in FPG between genders however, males had a slightly higher (+0.8%) mean FPG as compared to females (5.08 ± 1.2 mmol (95% CI [4.9–5.22] mmol) versus 4.97 ± 1.1 mmol (95% CI [4.8–5.1] mmol)). Pertaining to gender, the majority (87.6%) of female’s FPG was classified as optimal whilst the majority of male MAs also were classified as optimal FPG (75.1%). Only 4.0% of male MAs and 4.8% of female MA were classified as abnormal and suggestive of undiagnosed T2dm (Table 1). There was no relationship between participants’ age and FPG (*r* = .054, *P* = 0.24).

**Figure 3:**
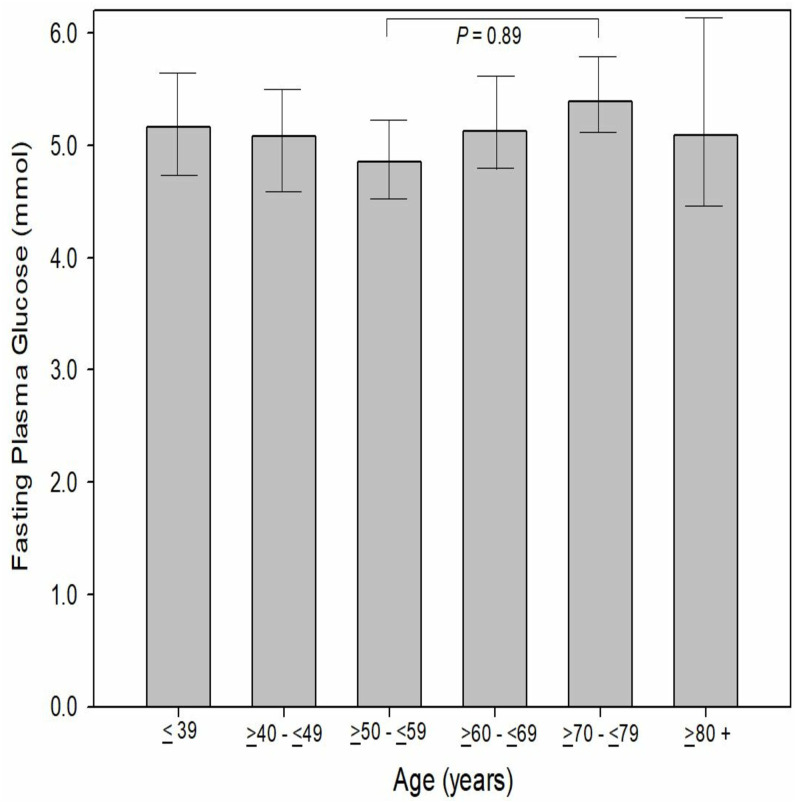
Participants fasting plasma glucose by decade of age.

**Figure 4:**
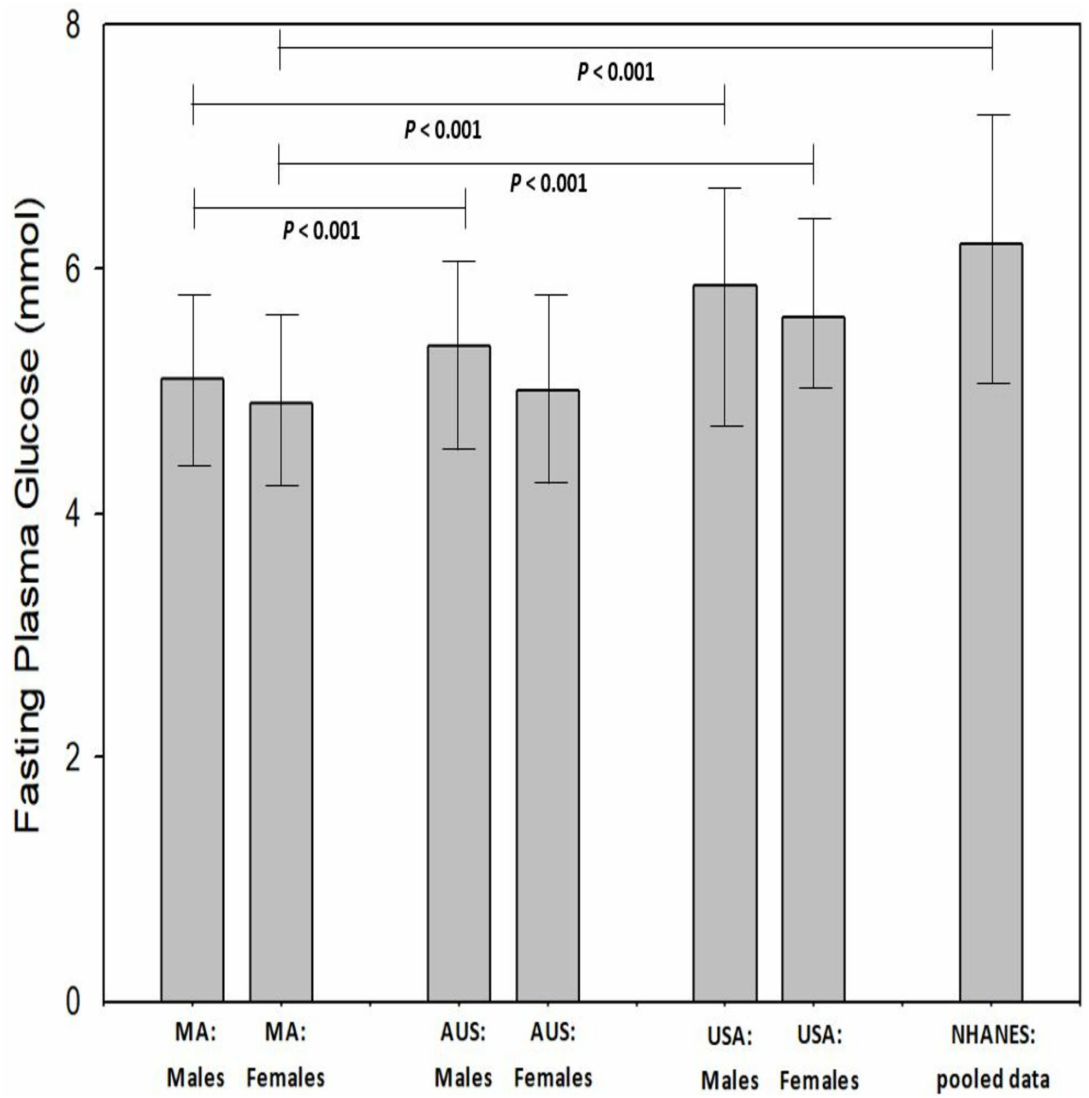
Masters athletes fasting plasma glucose compared to Australia General population, USA general population and National Health and Nutrition Examination Survey participants. Values are mean and 95% confidence intervals. Where: MA, masters athletes; AUS, general Australian population; USA, general United States population; NHANES, National Health and Nutrition Examination Survey.

When evaluated to age by decade, there was no significant difference (*P* = 0.09−1.00) between age by decade groups and FPG. The MAs aged between 50 to 59 (inclusive) reported the lowest FPG (4.85 ± 0.86 mmol, 95% CI [4.7–4.9]) while those MAs aged 70 to 79 years (inclusive) reported the highest mean FPG (5.39 ± 1.4 mmol, 95% CI [5.1–5.7]). In comparison to the Australian and United States general population (WHO data), male MAs had a significantly (*P* < 0.001) lower FGP (5.08 vs 5.37 vs 5.86 mmol, respectively). Female MAs has a significantly lower FPG than females from the United States (*P* < 0.001, 4.97 *vs* 5.60 mmol, respectively) however, only a slightly lower FPG than females from the Australian general population (−0.08%, *P* = 0.68, 4.97 *vs* 5.01 mmol) (Fig. 3). When MA FPG data (genders combines) was compared to the NHANES data set (mean 6.20 ± 1.9 mmol; 95% CI [6.1–6.3] mmol), MAs had a significantly (−23.1%, *P* < 0.001) lower FPG than the participants in the NHANES pooled data (Fig. 4).

Concerning risk factors for T2dm, a 12.6 percent of MAs reported a family history of type 2 diabetes mellitus (males 21.3% vs females 25.3%, *P* = 0.293). When compared as a subgroup, those individuals with no family history of T2dm has a slightly lower (−4.2%, *P* = 0.10) FPG than those MAs with a family history of T2dm (4.99 *vs* 5.20 mmol, respectively). Additionally, there was no difference between BMI classification groups and FPG (*P* = 0.27−1.00). There were, however, significant differences in FPG based upon the waist circumference classification. Those MAs with a gender-specific normal classification had a significantly lower (−9.2%, *P* = 0.009) FPG than those MAs with a high-risk waist classification. Additionally, those MAs with an at-risk waist classification, had a significantly lower (−8.3%, *P* = 0.049) FPG as compared to those MAs with a high-risk waist classification. There was no difference in smoking classifications and FPG (*P* = 0.964). Regarding resting BP classification and FPG, there was a significant difference identified (*P* = 0.045). Those MAs with a resting BP classification of normal had a lower (−11.2%, *P* = 0.054) than those MAs with a BP classification of Stage 2 hypertension (4.84 *vs* 5.38 mmol, respectively).

## Discussion

The primary aim of this study was to determine if hyperglycemia was present in male and female MAs who were participating in the WMG. Additionally, we sought to identify the glycaemia classifications, based upon the Royal Australian College of General Practitioners. Further, we also investigated if differences existed between the FPG level of MAs versus the general Australian and general US population. Masters athletes competing at the WMG who had FPG and related risk factors measurements (mass, BMI, resting blood pressure) recently attained from their general practitioner or primary care physician were included in this study. This study represents the largest sample of MAs to investigate glycemia and classify glycaemia into clinical categories.

We identified that MAs who participated in this study had clinically favorable FPG levels when compared to matched genders in the general Australian and US population. Specifically, male MAs had significantly (*P* < 0.001) lower FPG levels as compared to males in the general Australian and US populations. Female MAs had a significantly (*P* < 0.001) lower FPG than US females. Additionally, the MAs mean FPG was significantly (*P* < 0.001) lower than the mean values available in the NHANES data set (2017–2020 inclusive, *n* = 3,036).

The majority of MAs (group 75.5%, male MAs 71.8%, female MAs 80.3%) were classified as normal FPG as per the Royal Australian College of General Practitioners (Royal Australian College of General Practitioners, 2020). There were, however, a small percentage (4.3%) of MAs classified as T2dm likely (FPG of male (4.0%) and female (4.9%)) based upon their FPG level. The use of a single FPG test result to diagnose T2dm requires the presence of symptoms (polyuria, polydipsia, lethargy, blurred vision) (Royal Australian College of General Practitioners, 2020), which we did not investigate in this study. In comparison to the NHANES results (Centers for Disease Control, 2020), we identified the majority (50.7%) of their participants had elevated FPG indicating possible T2dm. However, approximately 14 percent of the NHANES participants were identified likely having T2dm. The high risk of T2dm in the NHANES participants is three-fold higher than observed in our MA participants.

Although there are no other MA studies available which have utilized the RACGP FPG classification system, the Australian Health Survey (Australian Bureau of Statistics, 2013) identified 3.39 percent of the participants (approximately 13,521,000) having a FPG greater ≥ 7.0 mmol. This value is similar to what we observed in our MAs (group 4.3%, male MAs 4.0%, female MAs 4.9%). With regard to FPG classified as prediabetes (≥5.5 to ≤6.9 mmol) 20.2 percent of the MAs were classified at this level (males 24.2% *vs* 14.8% females) whereas the Australian Health Survey participants had a slightly lower percentage (16.5%) classified as prediabetic. Unfortunately, the Australian Health Survey dataset contained only FPG and HbA1c results with no other outcome variables such as BMI, diet, physical activity/exercise or other risk factors for glycaemia. Therefore we are unable to make further comparisons to our risk factors assessed.

There is limited research on glycaemia in MAs. Climstein et al. (2011b) investigated chronic diseases in 216 male MAs who were participating in the GORF competition. In that study which featured only male MA participants, they also compared FPG between GORF participants based upon two age groups (<50 yrs and ≥50 yrs) and reported no difference (*P* = 0.682) between these two groups. The mean FPG value (5.74 ± 2.92 mmol) in the GORF participants was significantly higher (−14.8%, *P* < 0.001) than the mean FPG found in this study (5.0 ± 1.2 mmol). When comparing male MAs to the gender matched GORF participants, male MAs were found to have a significantly lower mean FPG (−12.9%, *P* < 0.001). Males in the GORF study were heavier (+15.9%) which may account for the higher mean FPG level, however the GORF participants were also younger (−12.2%). Regrettably, neither the GORF study nor the present study accounted for dietary influences which would impact fasting plasma glucose levels. For example, Ojo et al. (2018) reported in their meta-analysis that a low glycemic index (GI) diet resulted in a significant improvement in HbA1c (−0.5% absolute difference) and FPG (−6.59%). Their meta-analysis identified significant differences between low-GI diets and higher-GI diets on FPG levels (*P* < 0.0001). They concluded low GI-diets were more effective in controlling FPG than higher GI diets.

The progression from increased FPG levels to T2dm is well documented. Nichols, Hillier & Brown (2008) investigated over 46,000 individuals over three years who were not diabetic or had impaired fasting glucose. They found each 0.1 mmol increased the risk of developing T2dm by six percent. Further, those participants with a FPG between 5.0 and 5.2 mmol were 49 percent more likely to progress to T2dm than those participants with a FPG of <4.7 mmol. Bansal (2015) completed a review of the literature which included progression of impaired fasting glucose to T2dm. He reported the annual conversion rate from impaired fasting glucose to T2dm ranged from six to nine percent whereas the Diabetes Prevention Program Outcomes Study found the progression to be much higher at 11.0 percent (Knowler et al., 2009). The varying rates of progression in these studies was related to the FPG and other risk factors such as age, gender, ethnicity, SBP, HDL cholesterol and family history of T2dm (Bansal, 2015).

Our use of FPG as a biomarker for assessing risk of T2dm does have limitations such as the day-to-day viability. Ollerton et al. (1999) investigated the day-to-day variability of FPG in 193 newly diagnosed, non-medicated adults (mean age 54 ± 10 yrs) with T2dm. They reported the day-to-day variability in FPG was approximately 15 percent. The authors further identified that as the testing was conducted on two consecutive days, and diet and lifestyles changes (such as physical activity or exercise) were unlikely to have a significant effect the results. Therefore they attributed the majority (93%) of the variability identified to unexplained intraindividual biological factors, including the dawn phenomenon (O’Neal & Luther, 2022) where FPG is elevated in the early morning. Aside from the day-to-day variability associated with FPG as a biomarker for T2dm, the variability of FPG has also been identified as an independent risk factor for mortality. Wang et al. (2017) followed over 53,000 Chinese individuals in the general population over five years. They stratified FPG variability by quartiles and found when comparing the lowest quartile (<5.27 mmol) to the highest quartile (<13.11 mmol) that those individuals in the highest quartile had a 26 percent greater risk of developing cardiovascular disease and a 46 percent greater risk of all-cause mortality. Therefore, FPG as a biomarker provides clinical data beyond the risk of development T2dm.

With regard to risk factors for development of T2dm, our group has investigated the BMI of MAs and found it to be lower than the general Australian population. A literature review of 60 studies which included 10,788 MAs (Walsh, Heazlewood & Climstein, 2018) compared the BMI of MAs in different sports to various control groups. Walsh et al., reported the mean MAs BMI was 23.8 kg/m^2^ (±1.1) with a range from 20.8 kg/m^2^ in endurance runners to 27.3 kg/m^2^ in soccer players. Walsh and colleagues in their review identified the mean BMI was significantly lower (*P* < 0.001) than controls (−9.5%, 26.13 ± 1.7 kg/m^2^). These authors concluded that most, but not all studies included in the review, the BMI of MAs was significantly lower than controls and this afforded the MAs reduced risk with regard to a number of cardiometabolic diseases including T2dm.

Abdominal obesity, as measured by waist circumference, is recognized as being a stronger risk factor than overall obesity expressed as BMI for the future development of T2dm (Siren, Eriksson & Vanhanen, 2012). Siren and colleagues investigated the waist circumference of 715 men aged 40 years and older. They reported that a waist circumference of ≥ 94 cm was associated with a sensitivity for developing tT2dm of approximately 85 percent. In our study, 33.3 percent of male MAs were classified as high risk (*i.e.*, waist circumference ≥ 94 cm). Siren, Eriksson & Vanhanen (2012) concluded that a waist circumference ≥ 94 cm was the most predictive in identifying middle-aged men for developing T2dm. Feller, Boeing & Pischon (2010) also investigated waist circumference and risk for T2dm, however they incorporated BMI as a combined risk factor. They followed 960 individuals (583 male, 425 female) at eight years and found there was a significant interaction (*P* < 0.001) between BMI and waist circumference and the development of T2dm. Of interest, those individuals with a low or normal BMI with a large waist circumference had increased risk of developing T2dm. The relative risk ratio for males was 3.6 whereas slightly lower risk in females of 2.7. With regard to our MAs, only 8.1 percent met the criteria as investigated by Feller, Boeing & Pischon (2010), suggestive that a small percentage of our MAs at risk of future development of T2dm. The Epic-InterAct case-cohort study (Langenberg et al., 2012), reportedly the largest study of incident T2dm, was a multi-center trial involving 26 centers in eight European countries where they investigated the risk of developing T2dm in a cohort of 16,154 individuals. The investigators identified that waist circumference was independently and strongly associated with T2dm, particularly in women. Those women with a high BMI (≥35 kg/m^2^) and a high waist circumference were identified with a hazard ration of 31.8. In our MAs, only 10.6 percent of female participants met the Epic-InterAct criteria for a high BMI and high waist circumference.

Qiao & Nyamdorj (2010) conducted a review which included 52 studies (17 prospective and 35 cross-sectional) to determine whether BMI or waist circumference was a better predictor of T2dm. They identified mixed findings between the studies included in their review. For example, two of the prospective studies identified waist circumference to be a better indicator of predicting T2dm risk, however it was ethnicity specific (Mexicans, African Americans). Of the 35 cross-sectional studies, approximately one-third reported higher odds ratios associated with waist circumference as opposed to BMI. The authors concluded that both BMI and waist circumference were independently associated with risk of T2dm. Depending upon the BMI and/or waist circumference risk criteria utilized, a small percentage of our MA participants would be identified at risk for future development of T2dm.

Hypertension has previously been considered a comorbidity of T2dm. While elevated resting blood pressure in the upper range is not diagnostic of hypertension, under the new guidelines (Flack & Adekola, 2020) elevated blood pressure has been recognized as a risk factor for the development of T2dm (Mullican, Lorenzo & Haffner, 2009). Stahl et al. (2012) conducted a 35-year longitudinal study where they followed a random sample of 7,494 men aged 47 to 55 years. At follow-up, 13 percent of the men were diagnosed with T2dm, and the risk identified was found to increase based upon the resting SBP. For example, those men with a resting SBP of <130 mmHg had a 19 percent risk of developing T2dm whereas those men with a SBP ≥ 160 mmHg had a 49 percent risk of developing T2dm. When adjusted for age, BMI, cholesterol, smoking status and physical activity, the hazard ratio was 1.43 for those men with a resting SBP ranging from 130–139 mmHg and 1.95 for those men with a resting SBP ≥160 mmHg. The authors concluded that a high-normal SBP was shown to be a significant predictor of T2dm, independently of BMI and other T2dm risk factors. Using Stahl’s (Stahl et al., 2012) criteria, approximately 27 percent of our male MAs reported an elevated resting SBP between 130–139 mmHg (inclusive). However, only one male MA reported a resting SBP ≥ 160 mmHg. Based upon Stahl’s (Stahl et al., 2012) findings, approximately 28 percent of our male MAs would be at risk of developing T2dm. Kim et al. (2015) investigated the relationship of prehypertension and hypertension on the development of T2dm in 7,150 middle-aged Koreans (mean age 52 years, 52.5% female). At eight years follow-up they found that of all participants, approximately 15 percent developed T2dm. Specific to baseline resting blood pressure, approximately 11.2 percent of the participants who were normotensive, 17 percent of those pre-hypertensive and 22 percent of those hypertensive were found to have developed T2dm. Additionally, those middle-aged participants who were normotensive at baseline who developed pre-hypertension or hypertension had a 48 percent increased risk of developing T2dm.

The primary strength of this study was the sample size of 486 MAs participants, as the only previous published study investigating glycemia in MAs had a smaller sample (*n* = 216) which also only contained male MAs (Climstein et al., 2011a). An additional strength to our study was the use of pathology results for FPG whereas previous investigations have utilized hand-held glucometers (Yi et al., 2017) which have been shown to be less accurate. For example, Kermani et al. (2017) reported the sensitivity of three commercial glucometers ranged from 96 percent to 81 percent. Additionally, we requested participants attain recent physiological measures of height, mass, resting BP, waist circumference and FPG from their general practitioner or primary care physician. This requirement may explain why a small percentage (*n* = 8,070) of the WMG participants who responded to our survey participated in this aspect of the study (*n* = 486). Conversely, MAs (and/or their general practitioner) may perceive themselves as healthy and are not undergoing routine biochemical screenings and were therefore unable to provide the necessary data which included FPG.

As the aim of our study was to identify hyperglycemia in MAs as opposed to identifying T2dm we chose FPG as our primary outcome variable. Glycated hemoglobin (HbA1c) has been shown to be a better biomarker to identify T2dm (Sherwani et al., 2016) however, HbA1c is not a standard biomarker in individuals who are non-diabetic or at risk of T2dm and therefore this would have significantly reduced our sample size.

There were limitations to this study. Firstly, this was a cross-sectional rather than longitudinal study design. Our main aim was to identify MAs with hyperglycemia indicative of T2dm or at risk of developing T2dm, which we accomplished however, we are unable to determine what percentage of our participants will develop T2dm in the future. Although we were able to identify differences between MAs and our comparative populations, we were not able to investigate factors directly related to glycaemia such as diet and exercise as these confounding factors were not included in our survey. Also, participants were not required to upload pathology results to substantiate their data.

Although our MAs participant in this study presented with varying risk factors, including hyperglycemia, a large sample of MAs (n >8,000) were recently reported (Climstein et al., 2021) to have a significantly lower prevalence of a number of chronic conditions including T2dm as compared to the general Australian population (2.1% vs 5.1%, respectively).

The implications of our findings in this study indicate that MAs are indeed a model of successful ageing with regard to glycemia and other select related measures of risk. Future research, specifically longitudinal studies, are needed to determine the percentage and rate of progression of MAs to T2dm based upon glycemia, waist circumference, BMI and resting blood pressure.

## Conclusions

The primary aim of this study was to determine if hyperglycemia was present in the MAs participating in the WMG. This study represents the largest sample of MAs investigated in terms of investigating glycemia and classification of glycaemia into clinical categories, with a sample size more than double the sample size of previous work on hyperglycemia in MA. Additionally, our study included gender equity in the participant sample whereas the prior GORF study only contained only males.

Most, however not all, MAs were found to have normal glycaemia, with only a small percentage indicating a risk of developing T2dm (*i.e.*, impaired fasting glucose) and a smaller percentage identified with an abnormal FPG, suggestive of T2dm. Further, we also investigated if differences existed between the FPG level of MAs versus the general Australian and US populations. MAs who participated in this study had clinically favorable FPG levels compared to matched genders in the general Australian and US population with the majority of MAs classified as normal FPG. These findings suggest MAs participating in this study appeared to be at low metabolic risk for developing T2dm based upon FPG. The physical activity/exercise MAs complete may therefore be protective against hyperglycemia, at least while active as MAs. There were, however, a small percentage (4.3%) of MAs classified as T2dm likely based upon elevated FPG level.

Elevated resting blood pressure in the upper range, however not diagnostic of hypertension under the new guidelines, has been recognized as a risk factor for the development of T2dm. Using Stahl’s criteria (Stahl et al., 2012), approximately 28 percent of our male MAs would be at risk of developing T2dm based upon their reported resting blood pressure. The implications of our findings in this study indicate that MAs are indeed a model of successful ageing with regard to glycemia and other related outcomes measures. Future research, specifically longitudinal studies, are needed to determine the percentage and rate of progression of MAs to T2dm based upon glycemia, waist circumference, BMI and resting blood pressure. We therefore believe that the physical activity and exercise completed by MAs offers a protective metabolic effect, which unfortunately cannot be quantified by our study.

## Supplemental Information

10.7717/peerj.13389/supp-1 Supplemental Information 1 The data for outcome variables analyzed in this study

10.7717/peerj.13389/supp-2 Supplemental Information 2 STROBE checklist

## Additional Information and Declarations

### Data Availability

The following information was supplied regarding data availability: The raw data is available in the Supplementary File.
